# Boosting the free fatty acid synthesis of *Escherichia coli* by expression of a cytosolic *Acinetobacter baylyi* thioesterase

**DOI:** 10.1186/1754-6834-5-76

**Published:** 2012-10-11

**Authors:** Yanning Zheng, Lingling Li, Qiang Liu, Wen Qin, Jianming Yang, Yujin Cao, Xinglin Jiang, Guang Zhao, Mo Xian

**Affiliations:** 1 Qingdao Institute of Bioenergy and Bioprocess Technology, Chinese Academy of Sciences, Qingdao, 266101, China; 2 University of Chinese Academy of Sciences, Beijing, 100049, China; 3 College of Food Science, Sichuan Agricultural University, Yaan, 625014, China

**Keywords:** Thioesterase, *Acinetobacter baylyi*, *Escherichia coli*, Free fatty acid, Substrate specificity, Active-site residues

## Abstract

**Background:**

Thioesterases remove the fatty acyl moiety from the fatty acyl-acyl carrier proteins (ACPs), releasing them as free fatty acids (FFAs), which can be further used to produce a variety of fatty acid-based biofuels, such as biodiesel, fatty alcohols and alkanes. Thioesterases play a key role in the regulation of the fatty acid synthesis in *Escherichia coli*. Therefore, exploring more promising thioesterases will contribute to the development of industrial microbial lipids production.

**Results:**

We cloned and expressed a cytosolic *Acinetobacter baylyi* thioesterase (‘AcTesA) in *E. coli* by deleting its leader sequence. Protein sequence alignment, structure modeling and site-directed mutagenesis demonstrated that Ser^10^, Gly^48^, Asn^77^, Asp^158^ and His^161^ residues composed the active centre of ‘AcTesA. The engineered strain that overexpressed ‘AcTesA achieved a FFAs titer of up to 501.2 mg/L in shake flask, in contrast to only 20.5 mg/L obtained in wild-type *E. coli*, demonstrating that the expression of ‘AcTesA indeed boosted the synthesis of FFAs. The ‘AcTesA exhibited a substrate preference towards the C8-C16 acyl groups, with C14:0, C16:1, C12:0 and C8:0 FFAs being the top four components. Optimization of expression level of ‘AcTesA made the FFAs production increase to 551.3 mg/L. The FFAs production further increased to 716.1 mg/L by optimization of the culture medium. Fed-batch fermentation was also carried out to evaluate the FFAs production in a scaleable process. Finally, 3.6 g/L FFAs were accumulated within 48 h, and a maximal FFAs yield of 6.1% was achieved in 12–16 h post induction.

**Conclusions:**

For the first time, an *A. baylyi* thioesterase was cloned and solubly expressed in the cytosol of *E. coli*. This leaderless thioesterase (‘AcTesA) was found to be capable of enhancing the FFAs production of *E. coli*. Without detailed optimization of the strain and fermentation, the finally achieved 3.6 g/L FFAs is encouraging. In addition, ‘AcTesA exhibited different substrate specificity from other thioesterases previously reported, and can be used to supply the fatty acid-based biofuels with high quality of FFAs. Altogether, this study provides a promising thioesterase for FFAs production, and is of great importance in enriching the library of useful thioesterases.

## Background

Thioesterases remove the fatty acyl moiety from the fatty acyl-acyl carrier proteins (ACPs), releasing them as free fatty acids (FFAs). They play an essential role in chain termination during *de novo* fatty acid synthesis and have been proven to be important in fatty acid bioengineering [1]. Therefore, thioesterases are widely used for the microbial production of FFAs, which can be further applied to produce fatty acid-based biofuels, such as biodiesel, fatty alcohols and alkanes [2-5].

In addition, thioesterases from different organisms have varied substrate specificities, and can be used to tailor the composition of the FFAs. For examples, Cp FatB1 from *Cuphea palustris* has a substrate preference towards C8- and C10-ACPs, Uc FatB1 from *Umbellularia californica* prefers the C12-ACPs, and thioesterases from *Ricinus communis* and *Jatropha curcas* accumulated three major products, including C14, C16:1 and C16 straight chain FFAs [6-10].

In wild-type *E. coli*, the fatty acid biosynthesis was inhibited by fatty acyl-ACPs in the absence of phospholipids synthesis. Though not a few thioesterases have been reported to be capable of releasing the feedback inhibition of fatty acyl-ACPs, no extensive examination was carried out to test their abilities to produce FFAs in microbial cells. Only a few thioesterases were applied to overproduce FFAs [11-15]. Zhang *et al.* examined the effect of the overexpression of four different plant thioesterases on FFAs production of *E. coli*. Some of the thioesterases they examined were able to produce over 2.0 g/L FFAs, representing a strong ability of accumulating FFAs [10]. In addition, they also found that the level of FFAs production mainly depended on the acyl-ACP thioesterase employed [10]. Therefore, it is of great significance to find a promising thioesterase that has a strong ability of FFAs accumulation or a novel substrate specificity.

Above-mentioned thioesterases are all from plant sources. Little attention has been paid to bacterial thioesterases except the ‘TesA of *E. coli*. Many bacterial enzymes are superior in chemical production to their eukaryotic counterparts. For example, the mevalonate (MVA) production increased 50 folds by replacing the MVA upper pathway genes from *Saccharomyces cerevisiae* with those from *Enterococcus faecalis*[16]. In addition, their substrate specificities are probably quite different from the plant thioesterases so far reported. Therefore, more types of FFAs, which may contribute to improving the performance of fatty acid-derived biofuels, can be expected by expressing bacterial thioesterases. Though a bacterial thioesterase from *Streptococcus pyogenes* was employed for improving the fatty acid synthesis, expression of this thioesterase in *E. coli* only obtained 1.3-fold more total fatty acids than the wild-type *E. coli*, still with C16 and C18 fatty acids as its major components [17]. So this *S. pyogenes* thioesterase did not obviously enhance the production of FFAs. It again demonstrates that the thioesterase plays the key role in determining the amount and composition of FFAs. So it prompts us to discover some promising bacterial thioesterase genes for further improving the FFAs production.

The *Acinetobacter baylyi* thioesterase is expected to be functional in hydrolyzing fatty acyl-ACPs to FFAs, as *A. baylyi* naturally accumulates wax ester, whose formation requires the participation of FFAs [18,19]. But unfortunately, no investigation has been carried out to study the *A. baylyi* thioesterase thus far.

In this study, a thioesterase gene was cloned from *A. baylyi*, and was heterologously expressed in *E. coli* BL21(DE3). To investigate the enzymatic activity and substrate specificity of this thioesterase, its catalytic product was determined by gas chromatography. In addition, protein sequence alignment and structure analysis were carried out to elucidate its possible active centre, which was further determined by site-directed mutagenesis. The expression level of *A. baylyi* thioesterase and the fermentation medium were also optimized to further improve the production of FFAs. Finally, a fed-batch fermentation was performed to evaluate the FFAs production in a scaleable process.

## Results and discussion

### Sequence analysis

**Figure 1:**
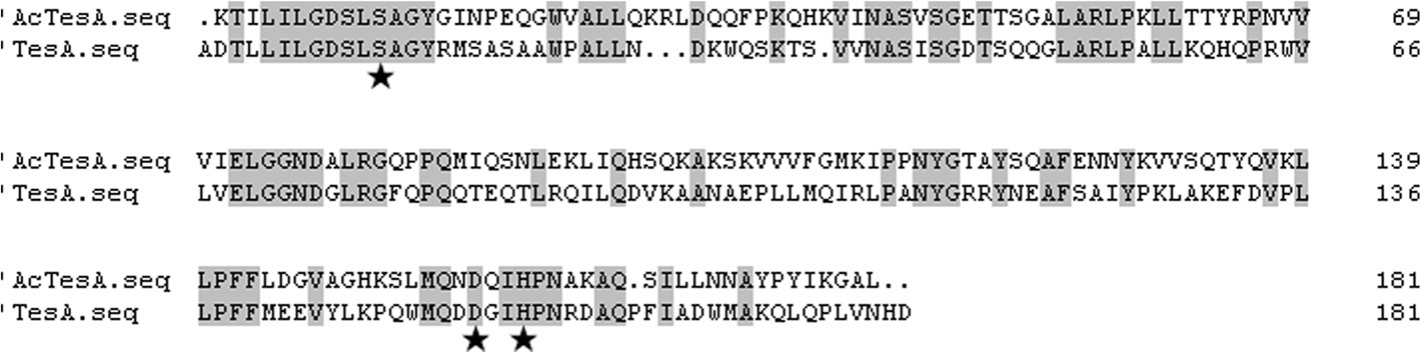
**Sequence alignment of deduced amino acids of the *A. baylyi *thioesterase (‘AcTesA) with the thioesterase I of *E. coli *(‘TesA).** The expressed ‘AcTesA has 37.97% amino acid sequence identity to the ‘TesA protein sequence, and shares the same catalytic-triad residues as ‘TesA, suggesting that ‘AcTesA may function as a SGNH-hydrolase. The grey backgrounds indicate the identical amino acid residues between ‘AcTesA and ‘TesA. The asterisk marked residues represent the speculated catalytic triad.

The *A. baylyi* thioesterase gene *AcTesA* contained an ORF of 636 bp. The AcTesA is predicted to possess a leader peptide at the N terminus by using the SignalP 3.0 Server, and the leader peptide will be cleaved between A30 and K31 in the conversion of precursor protein to mature protein [20,21]. The mature protein ‘AcTesA has 181 amino acids, with a calculated molecular weight of 19.9 kDa. The amino acid sequence alignment of *A. baylyi* thioesterase ‘AcTesA and *E. coli* thioesterase ‘TesA is shown in Figure 1, with identical amino acids are shaded in gray. The expressed ‘AcTesA has 37.97% amino acid sequence identity to the ‘TesA protein sequence. It was never reported previously that a thioesterase possesses such high amino acid sequence similarity to ‘TesA. The ‘AcTesA has low identities to plant thioesterases, such as 7.69% to AtFatA (*Arabidopsis thaliana*) and 8.12% to UcFatB1 (*Umbellularia californica*) [9,11]. This bacterial thioesterase has distant genetic relationship with the plant thioesterases, suggesting it may have much difference in substrate specificity with the plant ones.

Ser^10^-Asp^154^-His^157^ and Ser^10^-Gly^44^-Asn^73^ compose the catalytic triad and the oxyanion hole of ‘TesA, respectively [22-24]. The ‘AcTesA shares the same catalytic-triad residues as ‘TesA (Figure 1), suggesting that ‘AcTesA may function as a SGNH-hydrolase. The ‘AcTesA has a similar but not the same catalytic triad as the plant thioesterases [25], which use cysteine to compose their catalytic triad instead of serine. The plant thioesterases were still highly active when their cysteines were mutated to serines, while the thioesterases from rat and chicken livers retained up to 90% of the activities when their serines were substituted with cysteines [26-28]. These results demonstrated that the sulfhydryl group of cysteine and the hydroxyl group of serine were both able to nucleophilically attack of the substrates’ carbonyl carbon atom.

### Expression of recombinant ‘AcTesA protein in *E. coli* BL21(DE3)

**Figure 2:**
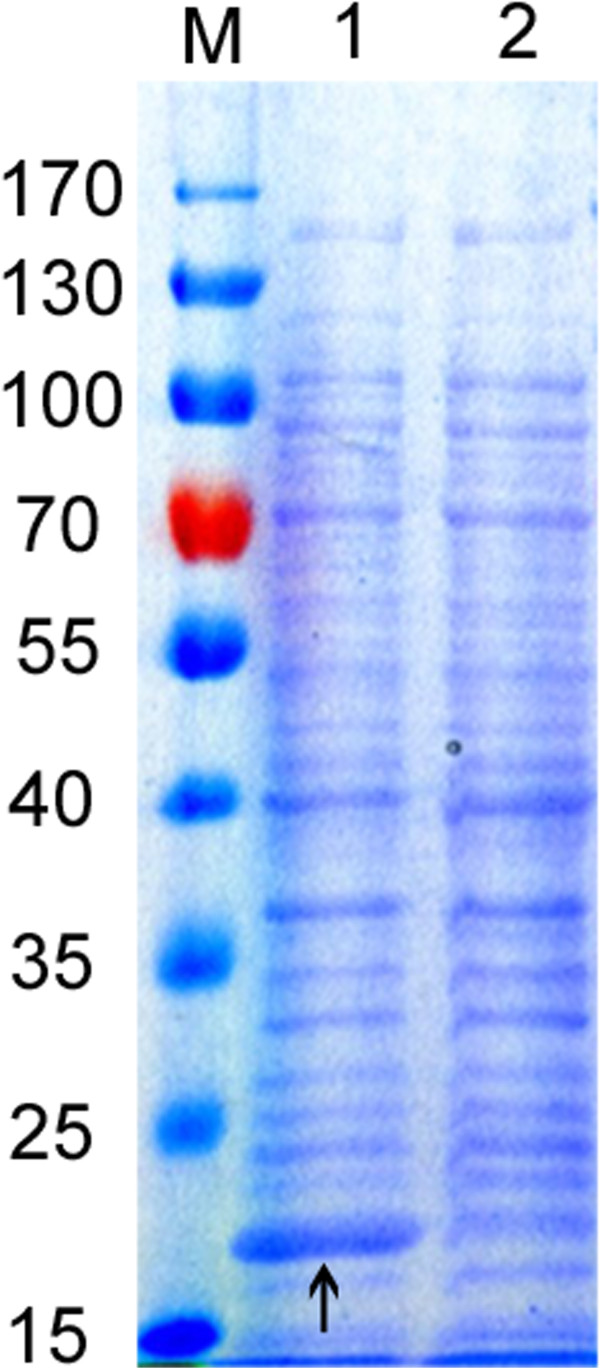
**SDS-PAGE analysis of recombinant proteins in crude extract from the engineered *E. coli *strain expressing *‘AcTesA *gene (LL8).***Lane M*, prestained protein molecular weight marker; *lane 1*, crude protein extracts from LL8 with IPTG induction; *lane 2*, crude protein extracts from LL8 without IPTG induction. The position corresponding to the overexpressed ‘AcTesA protein was indicated by an arrow.

It is of great importance to block the export of thioesterases to cellular periplasm, given they cleave the long chain acyl-ACPs substrates in the cytosol [29,30]. To trap the active enzyme within the cytosol, we deleted the leader sequence of the AcTesA, yielding ‘AcTesA. We next expressed ‘AcTesA in *E. coli* BL21(DE3), the resultant strain was designated as LL8. To confirm ‘AcTesA was correctly expressed in the cytosol, we carried out a SDS-PAGE analysis of the proteins in the cytosol of LL8. Figure 2 showed the gel electrophoresis patterns of samples analyzed with Coomassie brilliant blue stain (to visualize all proteins). We noted distinct bands of the expected size (19.9 kDa) from protein extracts of the induced LL8 strain compared with the uninduced control sample. It demonstrated ‘AcTesA was indeed soluble expressed in the cytosol of LL8. The soluble expression generally suggests the gene products are in their active form [31,32].

### Changes of the FFAs production by expressing *‘AcTesA*

To check if the expression of ‘AcTesA is capable of boosting the FFAs biosynthesis of *E. coli*, we determined the FFAs production in wild-type and engineered *E. coli* strains. In shake-flask experiments, the wild-type *E. coli* BL21(DE3) accumulated 20.5 mg/L FFAs, while LL8 produced ~25-fold more FFAs (501.2 mg/L) than BL21(DE3). It demonstrated that the activity of the endogenous thioesterase of wild-type *E. coli* was strictly regulated, which resulted in the tiny production of FFAs, and overexpression of ‘AcTesA could enhance the production of FFAs by releasing the feedback inhibition caused by fatty acyl-ACPs. The titer of 501.2 mg/L represents a high FFAs production achieved in shake flask.

**Figure 3:**
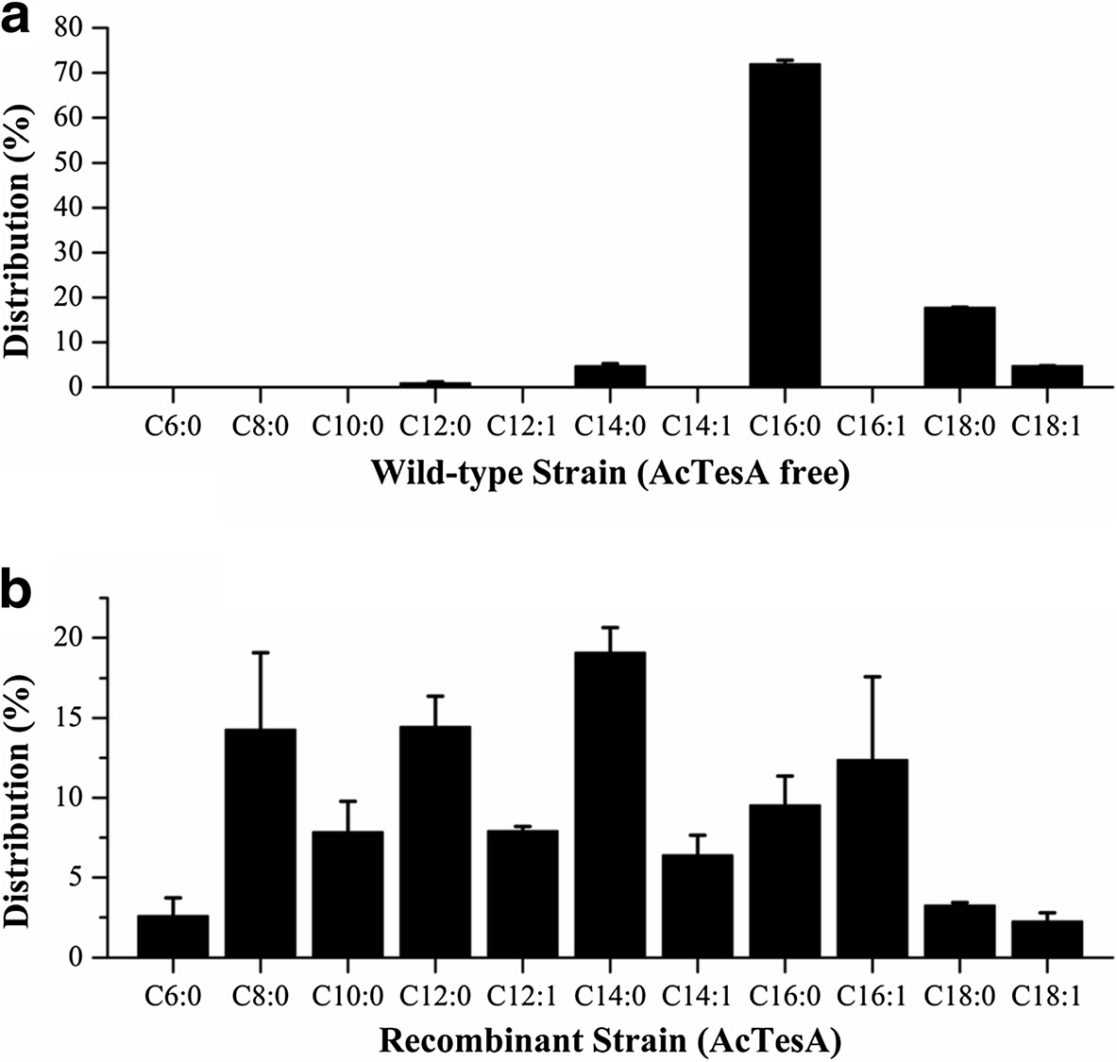
**The FFAs composition of the wild-type strain BL21(DE3) and the recombinant strain LL8.** The BL21(DE3) mainly produced the C16 and C18 FFAs, while the LL8 produced the FFAs ranging from C6 to C18, with C14:0, C12:0, C8:0 and C16:1 FFAs being the top four components. The unsaturated (C12:1, C14:1, C16:1 and C18:1) and shorter-chain FFAs (C6-C14) accounted for 87.2% of total FFAs produced.

The wild-type *E. coli* BL21(DE3) mainly produced the C16 and C18 FFAs, while LL8 greatly changed the composition of FFAs, accumulating a large proportion of C8-C14 FFAs (Figure 3). The LL8 produced the FFAs ranging from C6 to C18, with C14:0, C12:0, C8:0 and C16:1 components being the top four (Figure 3). It exhibits a quite different FFAs composition from other thioesterases previously reported [14]. As for the distribution of carbon chain length, C8, C12, C14 and C16 FFAs are the major components, accounting for 84.0% of total FFAs, and C6 (2.6%), C10 (7.9%) and C18 (5.5%) FFAs were the other FFAs observed. Compared with their saturated longer-chain counterparts (C16:0 and C18:0), the unsaturated (C12:1, C14:1, C16:1 and C18:1) or shorter-chain FFAs (C6-C14) are more suitable for the production of biofuels [11,15]. The unsaturated and shorter-chain FFAs LL8 synthesized accounted for 87.2% of total FFAs produced. Though Cao *et al.* also achieved high unsaturated fatty acid content (~70% of total fatty aicds) by expressing an *Arabidopsis thaliana* thioesterase (*AtFatA*) in *E. coli*, they only obtained a low titer of fatty acids [11,33]. These results indicated that *‘AcTesA* was a good candidate for the production of FFAs.

### Three-dimensional structure analysis of thioesterase ‘AcTesA

The ‘AcTesA from *A. baylyi* has the same catalytic-triad residues and oxyanion-related residues as the ‘TesA from *E. coli* (Figure 1). The mechanism for ‘TesA catalysis is that the hydroxyl group from the catalytic serine nucleophilically attack of the thioester bond of the substrate fatty acyl-ACPs, assisted by a histidine, which functions as an acid–base catalyst [22].

**Figure 4:**
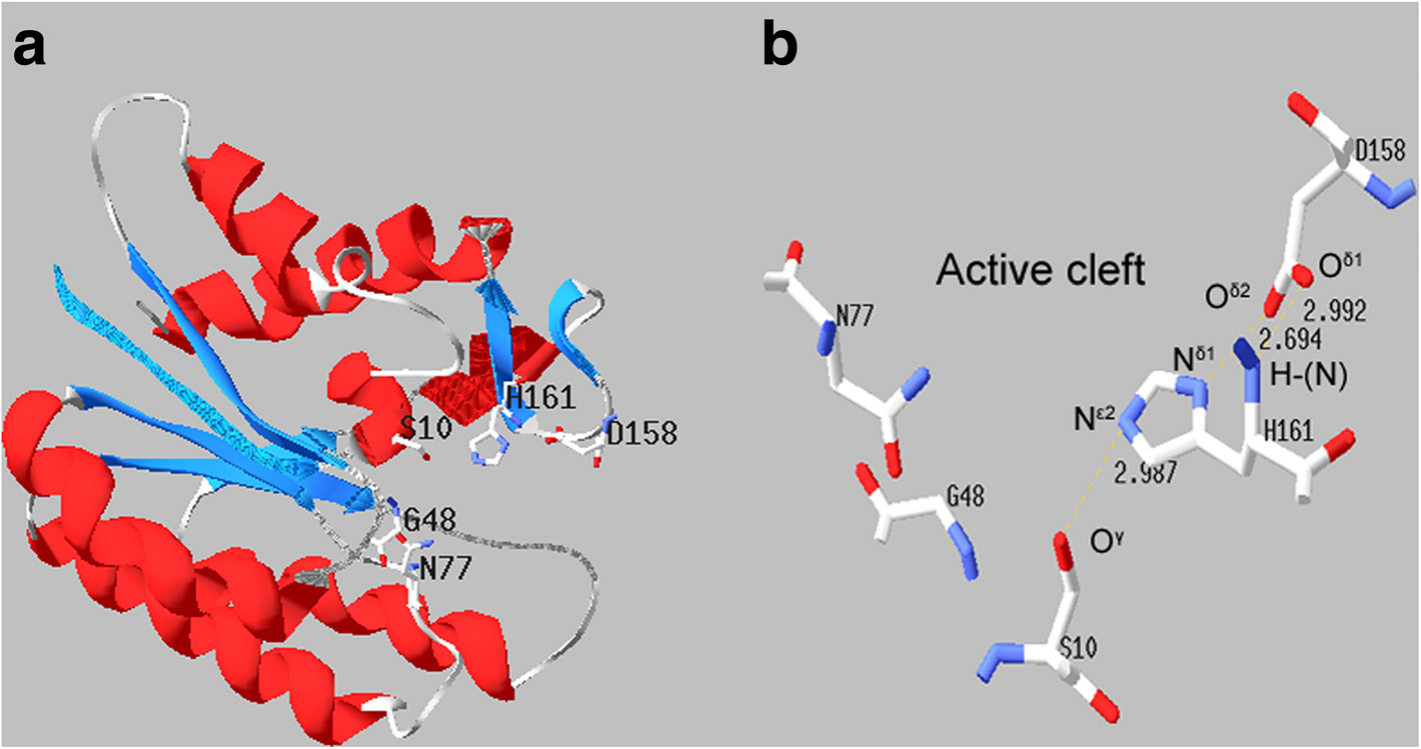
**Three-dimensional structure of the recombinant ‘AcTesA.a**) Overall structure of ‘AcTesA. The indicated residues are the catalytic triad, Ser^10^-Asp^158^-His^161^, and the stabilizing oxyanion residues, Gly^48^ and Asn^77^. **b**) Stereo view (Swiss-pdbviewer) of the catalytic triad and the oxyanion residues. The dashed lines indicate hydrogen bonding network, and the values represent the atom-atom distances.

To further elucidate the possible active centre of ‘AcTesA, the three-dimensional structure of the recombinant ‘AcTesA was generated using ‘TesA (PDB: 1IVN) as a template (Figure 4). The same as ‘TesA, the catalytic triad of ‘AcTesA, Ser^10^-Asp^158^-His^161^, are lined up in a row on one side of the active-site cleft. Ser^10^ takes the innermost position in the cleft, His^161^ locates in the middle, and Asp^158^ occupies the site nearest the surface of the protein (Figure 4a). Figure 4b shows that the distance between O^*δ*2^ of Asp158 and N^*δ*1^ of His^161^ is 2.694 Å. This allows the carboxylic group on Asp158 to form a low-barrier hydrogen bond with His^161^, increasing the pKa of its imidazole nitrogen. This then makes His^161^ act as a powerful general base. The hydrogen-bonding between the amide-N of His^161^ and the O^*δ*1^ of Asp^158^ may strengthen the coordination of the two amino acids. In addition, the N^*ε*2^ of His^161^ is close to O^*γ*^ of Ser^10^, with a distance of 2.987 Å. Therefore, the Ser^10^ can be deprotonated by His^161^. The deprotonated Ser^10^ serves as a nucleophile, attacking the carbonyl carbon of the fatty acyl-ACPs. The formed intermediate is stabilized by an oxyanion hole (Ser^10^-Gly^48^-Asn^77^). Collapse of this intermediate causes His^161^ to donate its proton to ACPs. The deprotonated His^161^ again obtained a proton from a water molecule, whose remaining OH^-^ attacks the carbonyl carbon of fatty acyl groups. Finally, the product FFAs are released, and the thioesterase restores its substrate-free confirmation after His^161^ donates a proton to Ser^10^.

### Site-directed mutagenesis

**Figure 5:**
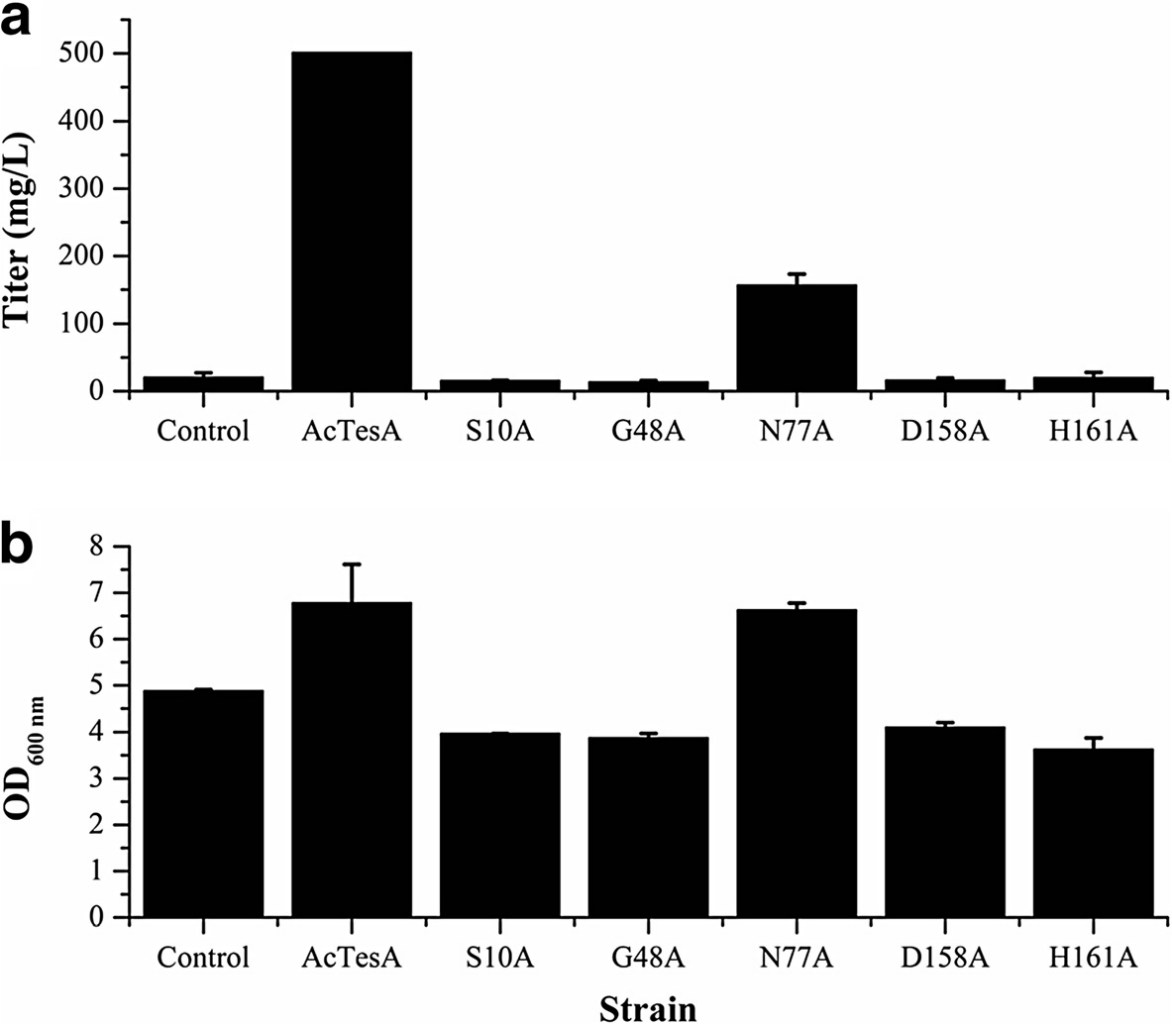
**Determination of the active-site residues of ‘AcTesA by site-directed mutagenesis.** FFAs productin (**a**) and cell densities (**b**) of the wild-type strain BL21(DE3) (control) and the recombinant strains (‘AcTesA, S10A, G48A, N77A, D158A and H161A). Compared with the native ‘AcTesA, the mutation to Ala at Ser^10^ (S10A), Gly^48^ (G48A), Asp^158^ (D158A) and His^161^ (H161A) residues led to totally loss of the thioesterase activity, and the mutation to Ala at Asn^77^ (N77A) residue caused the substantial decrease of the enzyme activity. The error bars represent the range from two independent experiments.

To further confirm Ser^10^, Gly^48^, Asn^77^, Asp^158^ and His^161^ are active-site residues, site-directed mutagenesis was carried out to convert each of these residues to an Ala. These mutants were expressed in *E. coli* and assayed for product synthesis. The resultant mutant strains S10A, G48A, D158A and H161A obtained the FFAs production less than 20 mg/L, as little as the titer attained by the wild-type BL21(DE3) (Figure 5a). In addition, the same as BL21(DE3), they only produced the C16 and C18 FFAs, without other types of FFAs being synthesized. The mutant strain N77A achieved an obviously higher FFAs titer (156.6 mg/L) than BL21(DE3), but the titer was still much lower than that achieved by LL8 (501.2 mg/L) (Figure 5a). These results demonstrate that mutation to Ala at Ser^10^, Gly^48^, Asp^158^ and His^161^ residues led to totally loss of the enzyme activity of ‘AcTesA, and the mutation to Ala at Asn^77^ residue caused the substantial decrease of the enzyme activity. The Ser^10^, Gly^48^, Asn^77^, Asp^158^ and His^161^ were thus validated to be the active-site residues of ‘AcTesA. Each of them either participates in the catalysis or refers to the substrate binding.

The Ser and His residues located at the active centre play a direct role in the catalysis. This also applies to plant thioesterases, given the enzyme will be completely inactivated if the cysteine or histidine in the catalytic triad is mutated to alanine [28]. Therefore, it is reasonable to believe that the bacterial and plant thioesterases utilize the uniform catalytic mechanism to hydrolyze the fatty acyl-ACPs.

In addition, all the strains had almost equivalent cell masses, except the strains expressing native ‘AcTesA and N77A mutant got slightly higher cell masses (Figure 5b). It demonstrated that the expression of recombinant thioesterases did not obviously affect the cell growth. The strains that accumulated more FFAs (‘AcTesA and N77A) even obtained the higher cell masses (Figure 5), suggesting the synthesized FFAs were not harmful to the *E. coli* cell.

### Optimization of the expression level of thioesterase ‘AcTesA

**Figure 6:**
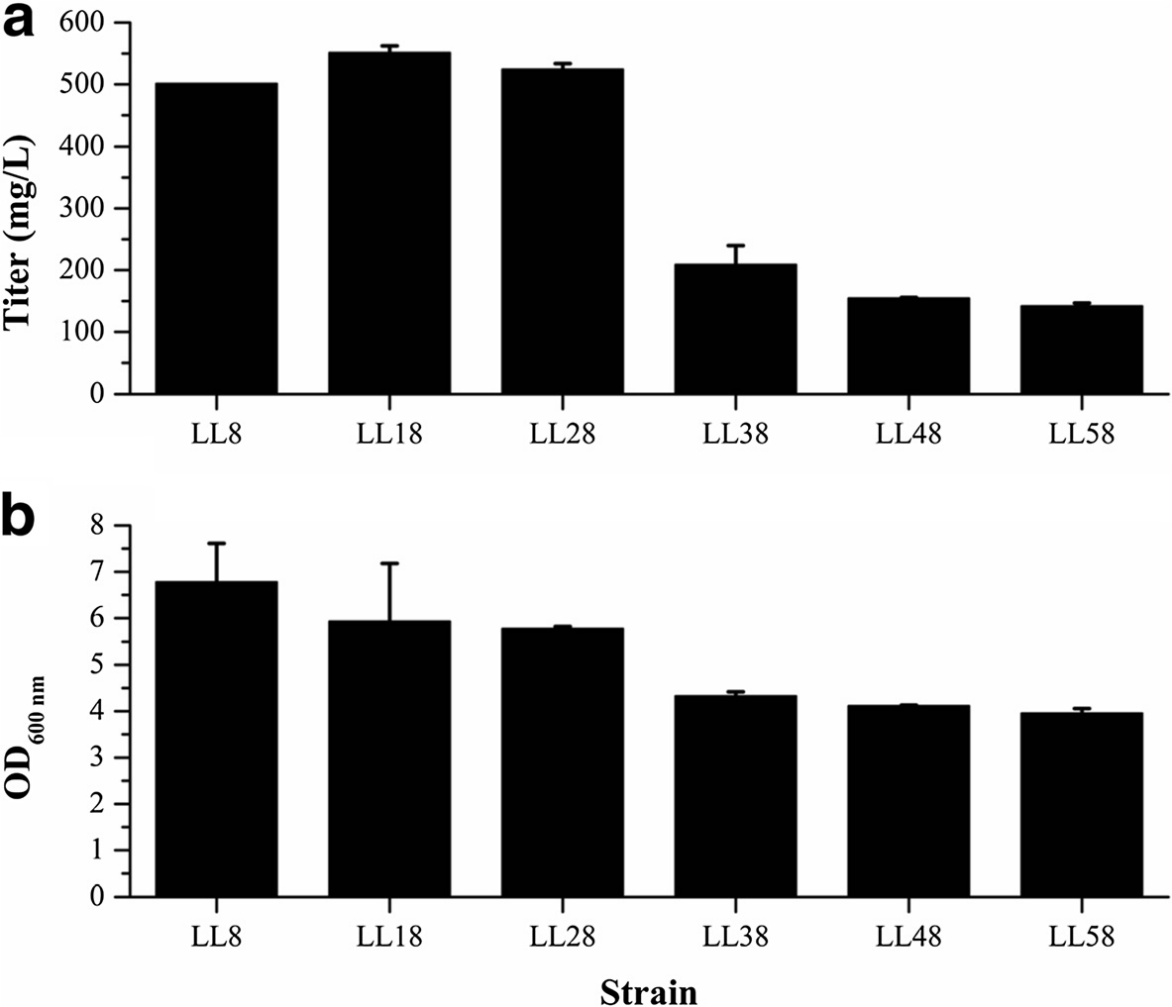
**Optimization of the expression level of ‘AcTesA.** The *‘AcTesA* under the control of T7 promoter (LL8, LL18 and LL28) achieved much higher FFAs production than it under the control of *ara*BAD promoter (LL38, LL48 and LL58). The strain LL18 using a medium copy number plasmid (pACYCDuet-1) achieved the highest FFAs production (551.3 mg/L).

To optimize the expression level of ‘AcTesA for higher FFAs production, plasmid vectors with different copy numbers and promoters were tested. The *‘AcTesA* under the control of T7 promoter (LL8, LL18 and LL28) achieved much higher FFAs production than it under the control of *ara*BAD promoter (LL38, LL48 and LL58). The strain LL18 using a medium copy number plasmid achieved the highest FFAs production (551.3 mg/L). The strains LL8 and LL28 obtained slightly lower titers of FFAs by using a lower and a higher copy number plasmids, respectively (Figure 6a).

Lennen *et al.* achieved a high production of fatty acids by using a medium-strength *ara*BAD promoter [34]. But in this study, a higher FFAs production was obtained by using a strong T7 promoter. It is worth mentioning that the T7 strains (LL8, LL18 and LL28) obtained higher cell masses than the *ara*BAD strains (LL38, LL48 and LL58) in the same sampling time (Figure 6b). It is probable that the lower cell masses resulted in the decreased FFAs production, and prolonged culture time may increase the FFAs production of the *ara*BAD strains to some extent. But generally speaking, the T7 strains achieved the higher productivities than the *ara*BAD strains. The high productivity is of great importance to decreasing the cost in mass production.

### Effect of carbon and nitrogen sources on FFAs accumulation

Glucose and glycerol are the most commonly used carbon sources. So the effect of glucose and glycerol on the FFAs production was firstly compared. Without addition of any organic nitrogen, LL18 produced 236.5 mg/L FFAs using glucose as the carbon source, while 222.9 mg/L using glycerol as the carbon source. This result demonstrates that both glucose and glycerol can be efficiently converted to FFAs by *E. coli*. This may be the reason why both the glucose and glycerol were widely used in the FFAs production [4,10,15,34].

**Figure 7:**
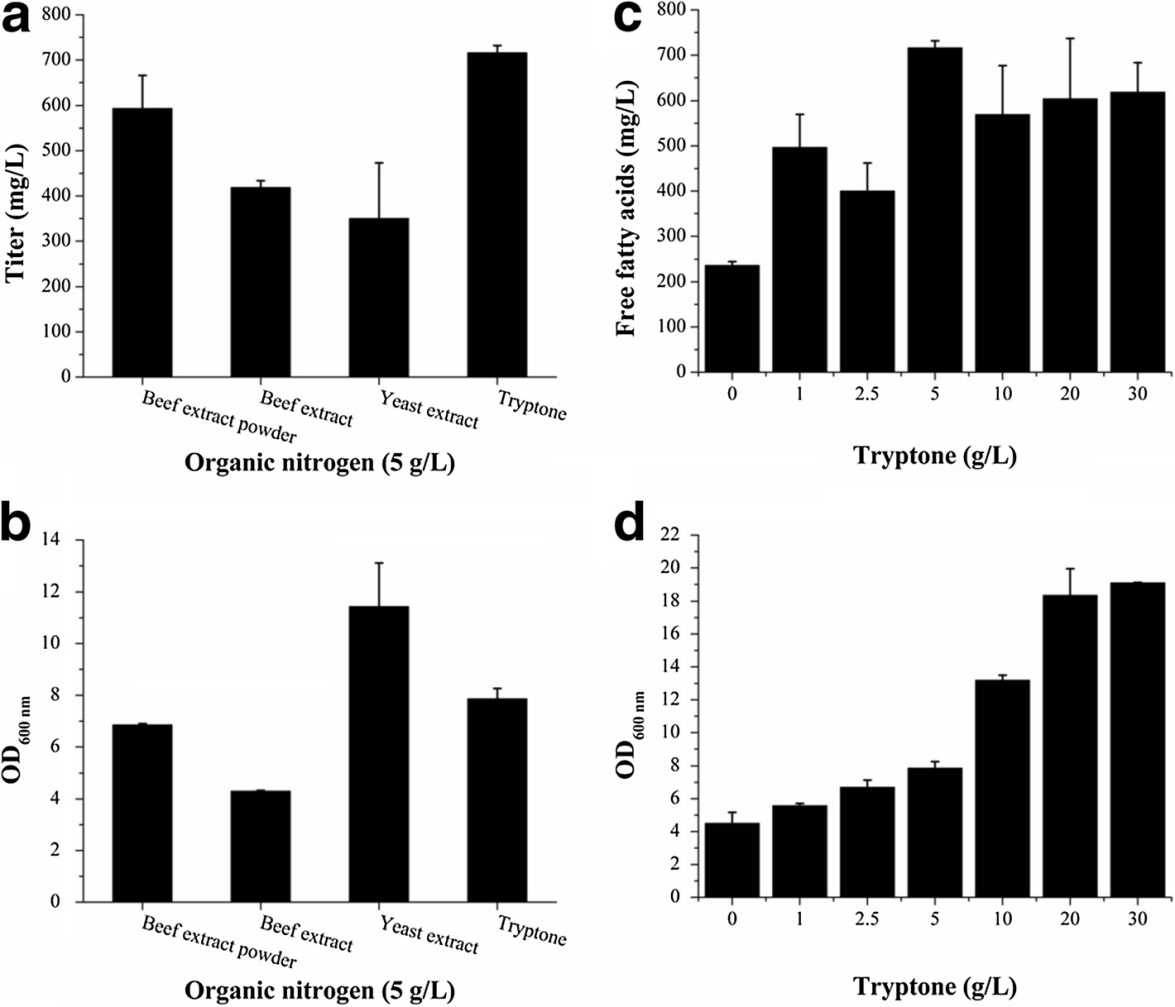
**Effect of nitrogen sources and contents on the production of FFAs.** The FFAs production (**a**) and cell densities (**b**) of the cultures supplemented with 5 g/L beef extract powder, beef extract, yeast extract and tryptone, respectively; The FFAs production (**c**) and cell densities (**d**) of the cultures supplemented with different contents of tryptone. The 5 g/L tryptone culture achieved the highest titer of FFAs (716.1 mg/L), with a medium cell density.

The source and content of the nitrogen in the medium play important roles in improving the biosynthesis of desired product [35]. In the previous experiments, 5 g/L beef extract powder was used as the organic nitrogen in the medium. To compare the effect of different organic nitrogen on the FFAs production, 5 g/L beef extract powder, beef extract, yeast extract and tryptone were added into the M9 medium as the organic nitrogen, respectively. Finally, the tryptone culture achieved the highest titer of FFAs (716.1 mg/L). The beef extract powder culture accumulated a slightly lower quantity of FFAs (593.7 mg/L) than the tryptone culture. The yeast extract culture accumulated the lowest titer of FFAs (350.3 mg/L), though it obtained a much higher cell mass than the other three cultures. The beef extract culture accumulated a comparable amount of FFAs (418.8 mg/L) with the yeast extract culture, with the lowest cell density (Figure 7a, Figure 7b).

Using tryptone as the organic nitrogen, the levels of organic nitrogen addition on the FFAs production were further tested. The LL18 was cultured in the media containing 0, 1, 2.5, 5, 10, 20 and 30 g/L tryptone, respectively, and its FFAs production in corresponding medium was determined. With elevated levels of tryptone, the obtained cell densities increased accordingly. The 5 g/L tryptone culture got a medium cell mass, but it achieved the highest FFAs production. The reduced tryptone addition led to decreased cell masses and FFAs production. The higher levels of tryptone contributed to the obviously increased cell masses, but resulted in decreased FFAs production (Figure 7c, Figure 7d).

The results demonstrate that the source and levels of organic nitrogen can greatly affect the FFAs production. The improved FFAs production in the medium containing 5 g/L tryptone mainly benefits from the modest cell growth and recombinant thioesterase (‘AcTesA) expression. The higher levels of organic nitrogen led to decreased FFAs production. The possible explanation is that the elevated levels of organic nitrogen resulted in the excessive quantities of functional thioesterase and thus initially rapider rates of FFAs accumulation, disturbing the cell viability and normal FFAs production of *E. coli*[34]. The lower levels of organic nitrogen lead to reduced cell masses and insufficient recombinant thioesterase, so the FFAs production also decreased.

### Fed-batch fermentation

**Figure 8:**
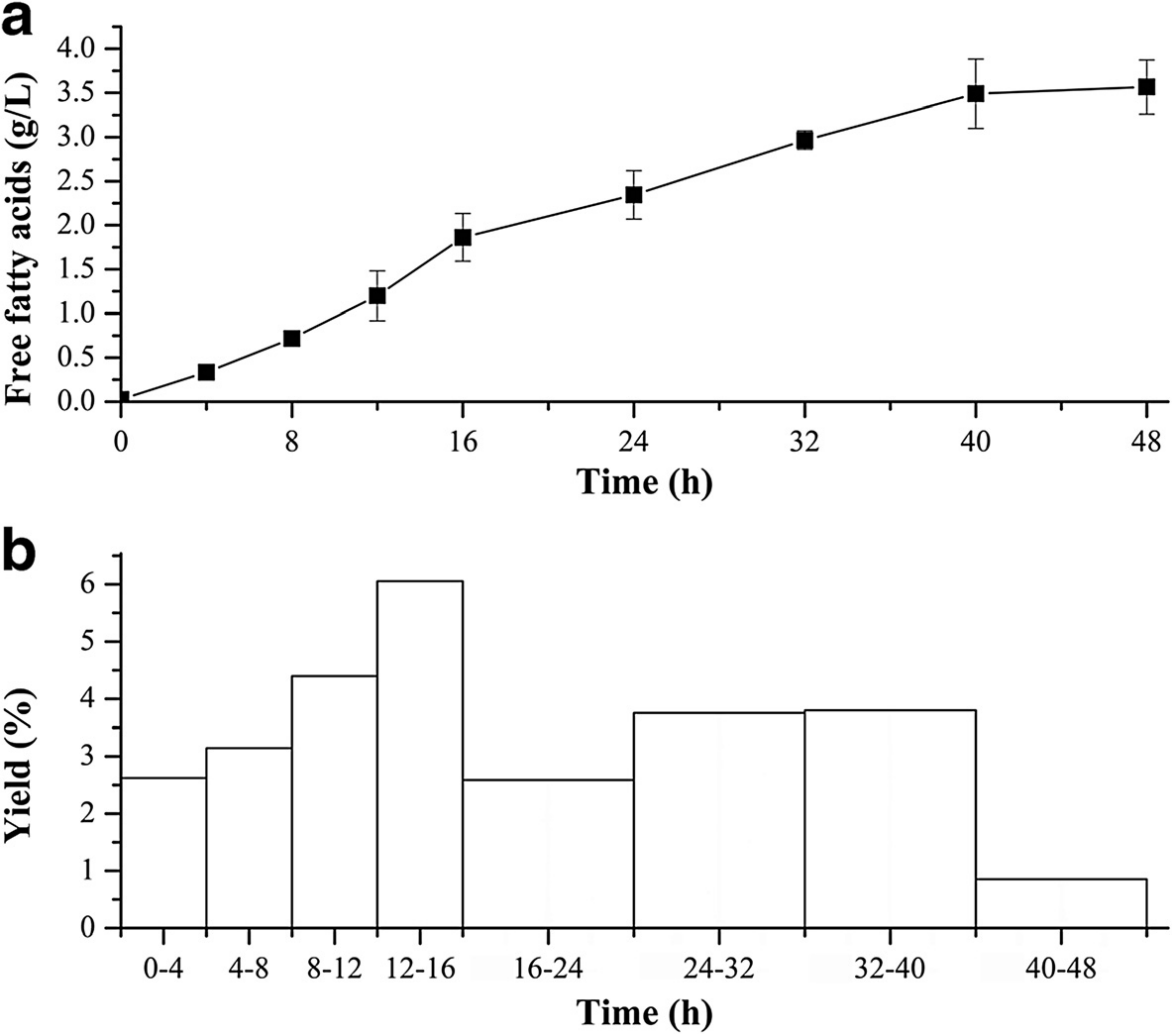
**Characterization of FFAs production of LL18 in fed-batch fermentation.a**) the FFAs production in fed-batch fermentation; **b**) the yield of LL18. LL18 finally accumulated 3.6 g/L FFAs, and achieved a maximal FFAs yield of 6.1% in 12–16 h post induction. The error bars represent the range from two independent experiments.

To evaluate the FFAs production in a scaleable process, fed-batch fermentations of LL18 was carried out at 5-L scale. At an OD_600nm_ of ~18, the culture was induced with 0.5 mM IPTG to induce the expression of the thioesterase [15]. The final OD_600nm_ at the end of the fermentation was ~30. Over the course of the fermentation, LL18 produced 3.6 g/L FFAs (Figure 8a). The FFAs productivity was relatively constant at 89 mg/L/h within 40 h. In addition, a maximal FFAs yield of 6.1% (i.e. 0.061 g FFAs /g glucose consumed) was observed in 12–16 h post-induction (Figure 8b).

Lu *et al.* optimized the FFAs-producing *E. coli* strain by detailed metabolic engineering, and they finally obtained 2.5 g/L FFAs in fed-batch fermentation [15]. In this study, without detailed optimization of the strain and fermentation, we achieved a FFAs titer of 3.6 g/L, ~1.5 fold higher than that obtained by Lu *et al.*[15]. It demonstrates that the ‘AcTesA is a quite promising thioesterase for the production of FFAs. The FFAs production can be further greatly improved by co-overexpressing the acetyl-CoA carboxylase to increase the supply of malonyl-CoA, an important precursor for fatty acid synthesis, and deleting the endogenous acyl-CoA synthetase to block the degradation of FFAs in a single host [15,36].

The FFAs yield of 6.1% is higher than the maximal yield of 4.8% obtained by Lu *et al*., but is still much lower than the theoretical maximal yield of ~30% (purely stoichiometric yield) [15]. A further improvement of FFAs production can be expected by efficient genetic reconstruction of the strain and detailed optimization of the fermentation conditions.

## Conclusions

For the first time, an *A. baylyi* thioesterase was solubly expressed in the cytosol of *E. coli* by deleting its leader sequence. This leaderless thioesterase (‘AcTesA) was discovered to be capable of greatly enhancing the FFAs production of *E. coli*. Without detailed optimization of the strain and fermentation, a encouraging titer of 3.6 g/L was finally achieved. In addition, it exhibited different substrate specificities from other thioesterases previously reported. The ‘AcTesA can be used to supply the biodiesel and chemical production with high quality of FFAs, and is of great importance in enriching the library of useful thioesterases. In the summary, our study provides a promising thioesterase for FFAs production.

## Methods

### Materials

*A. baylyi* ATCC 33305 was purchased from ATCC. *E. coli* BL21(DE3) were purchased from Invitrogen (Carlsbad, CA, USA). The expression vectors pCOLADuet-1, pACYCDuet-1 and pET-28a(+) were purchased from Novagen (Darmstadt, Germany). The expression vectors pBAD18, pBAD30 and pBAD33 were originally constructed by Guzman *et al*. [37]. All restriction enzymes and T4 DNA ligatase were purchased from Fermentas (Vilnius, Lithuania). The PrimeSTAR HS DNA polymerase was supplied by Takara Biotechnology (Dalian, China). Oligonucleotides were ordered from Generay Biotechnology (Shanghai, China). The arachidic acid was ordered from Alfa Aesar (Ward Hill, MA).

### Plasmid construction

The thioesterase gene ‘*AcTesA*, a leaderless version of *AcTesA* (GeneID: 2881191), was PCR-amplified from genomic DNA of *A. baylyi* ATCC 33305 with the primer set AcTesA-NcoF and AcTesA-BamHR, and inserted into the *NcoI*/*BamHI* sites of pCOLADuet-1, pACYCDuet-1 and pET-28a(+) to give pLL8, pLL18 and pLL28, respectively. In order to yield pLL38, pLL48 and pLL58, the ‘*AcTesA* gene was firstly PCR-amplified with the primer set rAcTesA-SacF and AcTesA-HindR, and then inserted into the *SacI*/*HindIII* sites of pBAD18, pBAD30 and pBAD33, respectively. All plasmids used in this work are listed in Table 1, and the primers used to amplify the ‘*AcTesA* gene are listed in Table 2.

**Table 1:** Bacterial strains and plasmids used in this study

**Plasmid or strain**	**Relevant genotype**	**Reference or source**
Plasmids		
pET-28a(+)	pBR322 *ori lacI* T7*lac* Kan^r^	Novagen
pACYCDuet-1	P15A *ori lacI* T7*lac* Cm^r^	Novagen
pCOLADuet-1	ColA *ori lacI* T7*lac* Kan^r^	Novagen
pBAD18	pBR322 *ori ara*BAD Amp^r^	37
pBAD30	pACYC184 *ori ara*BAD Amp^r^	37
pBAD33	pACYC184 *ori ara*BAD Cm^r^	37
pLL8	pCOLADuet-1 harboring *‘AcTesA* from *A. baylyi*	This study
pLL18	pACYCDuet-1 harboring *‘AcTesA* from *A. baylyi*	This study
pLL28	pET-28a(+) harboring *‘AcTesA* from *A. baylyi*	This study
pLL38	pBAD18 harboring *‘AcTesA* from *A. baylyi*	This study
pLL48	pBAD30 harboring *‘AcTesA* from *A. baylyi*	This study
pLL58	pBAD33 harboring *‘AcTesA* from *A. baylyi*	This study
pS10A	pCOLADuet-1 harboring *‘AcTesA* with a mutation to Ala at Ser^10^ residue	This study
pG48A	pCOLADuet-1 harboring *‘AcTesA* with a mutation to Ala at Gly^48^ residue	This study
pN77A	pCOLADuet-1 harboring *‘AcTesA* with a mutation to Ala at Asn^77^ residue	This study
pD158A	pCOLADuet-1 harboring *‘AcTesA* with a mutation to Ala at Asp^158^ residue	This study
pH161A	pCOLADuet-1 harboring *‘AcTesA* with a mutation to Ala at His^161^ residue	This study
Strains		
BL21 (DE3)	F^-^*ompT gal dcm lon hsdSB(r*_*B*_^*-*^*m*_*B*_^*-*^*)* λ(DE3)	Invitrogen
LL8	*E. coli* BL21 (DE3) bearing pLL8	This study
LL18	*E. coli* BL21 (DE3) bearing pLL18	This study
LL28	*E. coli* BL21 (DE3) bearing pLL28	This study
LL38	*E. coli* BL21 (DE3) bearing pLL38	This study
LL48	*E. coli* BL21 (DE3) bearing pLL48	This study
LL58	*E. coli* BL21 (DE3) bearing pLL58	This study
S10A	*E. coli* BL21 (DE3) bearing pS10A	This study
G48A	*E. coli* BL21 (DE3) bearing pG48A	This study
N77A	*E. coli* BL21 (DE3) bearing pN77A	This study
D158A	*E. coli* BL21 (DE3) bearing pD158A	This study
H161A	*E. coli* BL21 (DE3) bearing pH161A	This study

**Table 2:** Primers used in this study

**Name**	**Sequence (5’**→**3’)**
AcTesA-NcoF	CTAGCCATGGGCAAAACCATTCTTATCTTAGGCG
AcTesA-BamHR	CAGGGATCCTTATAAAGCGCCTTTAATATATGG
rAcTesA-SacF	GACGAGCTCAGGAGGTAAAAAAACATGGGCAAAACCATTCTTATCTTAGGCG
AcTesA-HindR	CTACCAAGCTTTTATAAAGCGCCTTTAATATATGG
S10A-F	CTTATCTTAGGCGACGCTCTGAGTGCGGGTTATGG
S10A-R	CTCAGAGCGTCGCCTAAGATAAGAATGGTTTTGCC
G48A-F	GCCAGTGTAAGTGCGGAAACCACCAGTGGTGC
G48A-R	GTTTCCGCACTTACACTGGCATTAATGACTTTATG
N77A-F	GAGCTTGGTGGTGCTGATGCATTAAGAGGAC
N77A-R	GCATCAGCACCACCAAGCTCAATGACCACCAC
D158A-F	CTAATGCAAAATGCCCAGATCCATCCAAATGC
D158A-R	CTGGGCATTTTGCATTAGACTTTTGTGTCCAG
H161A-F	CAAAATGACCAGATCGCTCCAAATGCCAAAGCCCAG
H161A-R	CATTTGGAGCGATCTGGTCATTTTGCATTAGACTTTTG

Underlines indicate restriction enzyme sites or mutagenized codons.

### SDS-PAGE analysis of recombinant protein

*E. coli* BL21(DE3) harboring pLL8, namely LL8 strain, was cultivated in LB medium supplemented with kanamycin at 37°C until its OD_600nm_ reached 0.6~0.8. Then 0.5 mM isopropyl β-D-thiogalactoside (IPTG) was added and the culture was switched to grow at 30°C for 6 h. The cells were first harvested by centrifugation at 10,000 g for 2 min, resuspended with 0.05 M sodium phosphate buffer (pH 7.4) after washed twice with the same buffer, and finally disrupted by sonication. The resultant supernatant by centrifuging for 30 min at 13000 g was collected for SDS-PAGE analysis.

### Structure modeling

The models of the theoretical ‘AcTesA structures were built on a public website Swiss-Model (http://swissmodel.expasy.org), using the thioesterase I of *Escherichia coli* (PDB ID: 1IVN) as a template.

### Site-directed mutagenesis

A method based on the amplification of the entire plasmid using primers that include the desired changes was employed for the site-directed mutagenesis [38]. The PrimeSTAR HS DNA polymerase (Takara) was used for the PCR. The Ser-10, Gly-48, Asn-77, Asp-158 and His-161 of the thioesterase ‘AcTesA were separately mutated to Ala by using the mutant primers S10A-F/S10A-R, G48A-F/G48A-R, N77A-F/N77A-R, D158A-F/D158A-R and H161A-F/H161A-R, respectively (Table 2).

### Bacterial strains, media, and growth conditions

The bacterial strains used in this study are listed in Table 1. *E. coli* BL21(DE3) (Invitrogen, Carlsbad, CA) was used as the host to overproduce proteins. During strain construction, cultures were grown aerobically at 37°C in Luria Broth (10 g/L tryptone, 10 g/L NaCl, and 5 g/L yeast extract). Kanamycin (50 mg/L), Ampicillin (100 mg/L) or chloramphenicol (34 mg/L) was added if necessary. For initial production experiments in shake flasks, strains were grown in a M9 medium (37.8 g/L Na_2_HPO_4_·12H_2_O, 7.5 g/L KH_2_PO_4_, 1 g/L NH_4_Cl, 0.5 g/L NaCl, 4 mM MgSO4) containing 20 g/L of glucose or 20 g/L of glycerol. The engineered strains were fed with glycerol as carbon source if they carried the recombinant plasmids using *ara*BAD promoter, otherwise they were fed with glucose. In addition, beef extract powder, beef extract, yeast extract or tryptone was added into the M9 medium if necessary. The media contained 5 g/L beef extract powder if there was no specific explanation. Protein production was induced with 0.5 mM isopropyl β-D-thiogalactoside (IPTG) at 30°C, and the cultures were harvested after 40 h post induction.

The fed-batch fermentation was carried out in a 5 L BIOSTAT® B plus fermentor (Sartorius Stedim Biotech GmbH, Goettingen, Germany). The strain was grown in the M9 medium supplemented with 5 g/L tryptone. The fermentation temperature was controlled at 30°C and the pH at 7.0. The pH was maintained using NH_3_·H_2_O. Cells were induced at an OD_600nm_ of ~18 using 0.5 mM IPTG. The glucose feed solution was continuously added into the cultures at the rates of 2~3 g/L/h.

### Detection of FFAs

The harvested cultures were firstly adjusted to pH 2.0 with 1 M HCl. The arachidic acid, which was dissolved in chloroform, was then added into the acidified cultures and used as the internal standard. An equal volume of chloroform–methanol (v/v, 2:1) with the culture was next added to extract the lipids [36]. The resultant blends were vortexed for a few minutes and then left overnight. The FFAs were directly quantified without derivatization by a gas chromatograph (GC) equipped with a flame ionization detector (FID) [39]. The system consisted of model 450-GC (Varian, Walnut Creek, CA) and a model 8400 AutoSampler (Varian). The separation of FFAs was performed using a CP-FFAP CB capillary column (25 m×0.25 mm; 0.2 μm film thickness) purchased from Agilent Technologies. The oven temperature was initially held at 100°C for 1 min, then raised with a gradient of 10°C/min until reaching 250°C, and finally held for 10 min. Nitrogen was used as the carrier gas. The injector and detector were held at 270°C and 300°C, respectively.

## Abbreviations

ACP: Acyl carrier protein; CoA: Coenzyme A; FFAs: Free fatty acids; IPTG: Isopropyl β-D-thiogalactoside; PCR: Polymerase chain reaction; GC: Gas chromatography; FID: Flame ionization detector.

## Competing interests

The authors declare that they have no competing interests.

## Authors’ contributions

YZ, LL, WQ, GZ and MX designed the research and prepared the manuscript. YZ, LL and QL did the lab work, plasmid construction, site-directed mutagenesis, strain cultivation and product detection. JY, YC and XJ helped to construct the plasmid and perform the fed-batch fermentation. All authors read and approved the final manuscript.
